# Alcohol diluent provides the optimal formulation for calcium chloride non-surgical sterilization in dogs

**DOI:** 10.1186/s13028-014-0062-2

**Published:** 2014-10-14

**Authors:** Raffaella Leoci, Giulio Aiudi, Fabio Silvestre, Elaine A Lissner, Giovanni M Lacalandra

**Affiliations:** Department of Emergency and Organ Transplantation (DETO), Section of Veterinary Clinic and Animal Production, University of Bari Aldo Moro, SP per Casamassima km 3, Valenzano, BA Italy; Parsemus Foundation, PO Box 2246, Berkeley, CA 94702 USA

**Keywords:** Calcium chloride, Canine, Chemical castration, Dog, Nonsurgical sterilization, Population management

## Abstract

**Background:**

Surgical castration is widely used to sterilize male dogs, but has significant impacts on time to perform the operation, recovery of the animals as well as cost, which can limit population control programs. Previous research has shown intratesticular injection of calcium chloride dihydrate (CaCl_2_) in saline to be a promising alternative to surgery. However, long-term azoospermia was not maintained at dosages low enough to avoid side effects. In the search for an optimized formulation, the current investigation is the first study on long-term sterilization effects of intratesticular injection of CaCl_2_ in either lidocaine solution or alcohol in dogs. CaCl_2_ at 20% concentration in lidocaine solution or alcohol was administered via intratesticular injection to groups of 21 dogs each. The treated animals were examined at 2, 6, and 12 months for sperm production, blood levels of testosterone, and side effects; at time zero and 12 months for testicular size and semen volume. The experimentally treated animals were compared to a control group receiving saline injection only.

**Results:**

Testicles of dogs treated with CaCl_2_ in either diluent significantly decreased in size. After administration of CaCl_2_ in lidocaine solution, sterility was achieved for at least 12 months in 75% of treated dogs. However, optimal long-term contraceptive effectiveness was achieved with CaCl_2_ in alcohol, which resulted in azoospermia over the 12-month study period. Testosterone levels significantly decreased following treatment with CaCl_2_, and sexual activity disappeared. Although testosterone returned to baseline levels by 12 months for the group treated with CaCl_2_ in lidocaine, dogs injected with CaCl_2_ in alcohol had a 63.6% drop in testosterone level, which remained at the low end of physiological range throughout the study. No adverse effects were noted.

**Conclusions:**

A single, bilateral intratesticular injection of 20% CaCl_2_ in 95% ethanol was a reliable method for induction of sterilization in 18–28 kg male dogs in this study. The approach showed long-term efficacy and reduced sexual behavior. This chemical method of sterilization might provide an effective, efficient alternative to surgical castration that can have positive impacts on dog welfare.

## Background

Canine overpopulation remains a problem facing many countries throughout the world. Alternative methods to surgical sterilization that are effective, easy to administer, safe, and affordable would offer immense benefits, allowing animal welfare organizations, public health programs, and governments to reach further with limited resources [1].

An intratesticular injection of calcium chloride dihydrate (CaCl_2_) in solution represents a promising method for non-surgical sterilization [2-7]. A previous dose-determination study reported that a 20% solution of CaCl_2_ in saline demonstrated good long-term efficacy without the undesirable side effects that occurred with higher dosages [2]. These findings partially confirmed the results of short-term, histology-based studies on CaCl_2_ by other investigators who used a 20% concentration [3,5-7]. However, when 20% CaCl_2_ in saline solution, as typically used for sterilization, was evaluated for efficacy over a longer period, the effect was not permanent: sperm production returned in some of the treated dogs, and testosterone levels increased to baseline levels by 12 months following injection [2]. For CaCl_2_ to be used effectively for canine sterilization, the formulation must be optimized to ensure permanent azoospermia.

The effectiveness of CaCl_2_ as a sterilizing agent may be augmented by the diluent used in the formulation. The earliest published abstract on the use of intratesticular injection of CaCl_2_ for sterilization in a variety of animals reported that aqueous solutions permitted higher concentrations, but tinctures in 80%-99% alcohol had the advantages of less pain, less peripheral inflammation, and more consistent results [8]. Intratesticular administration of a tincture of CaCl_2_ in ethanol in dogs was reported to have anesthetic properties, in comparison with saline solutions of CaCl_2_ [4]. Also, alcohol is known to have independent utility as a sterilant. In 1998, Yoon and Yoon [9] found that chemical castration with alcohol alone was as effective as orchiectomy in reducing testosterone levels in blood of rats. Furthermore, a single injection of 95% ethanol directly into the *vas deferens* caused atrophy of the *cauda epididymis*. Extensive necrosis and exfoliation of the seminiferous elements were conspicuous [10], with irreversible obstructive necrosis [11,12].

In addition, solutions of CaCl_2_ in other diluents have been used. Samanta and Jana reported on the effectiveness of lidocaine derivatives as diluents for CaCl_2_ chemosterilization in dogs and cats [5-7]. For example, using 1% lignocaine hydrochloride as a base for intratesticular injections of CaCl_2_ in dogs resulted in complete degeneration of germ cells in a 45-day trial [6]. The investigators reported that these changes may have been due to the necrotizing properties of CaCl_2_ and/or the significant reduction in intratesticular and blood levels of testosterone.

Despite the promising results on the use of CaCl_2_ as a nonsurgical sterilization method, little is known about long-term effectiveness or impact on dog health and behavior. This lack of information has hampered the widespread application of CaCl_2_ to address the problem of dog overpopulation.

The objective of the current study was to evaluate the long-term (i.e., 1 year) efficacy of intratesticular injection of 20% CaCl_2_ in alcohol versus lidocaine for the relative ability to halt sperm production and reduce blood levels of testosterone in dogs. We hypothesized a greater effectiveness for one or both formulations, as compared to historical use of 20% CaCl_2_ in saline alone.

## Methods

### Animals

For the study, 52 healthy, owned, mixed-breed male dogs living in a shelter were selected. The dogs were 2 to 6 years of age (mean = 3.5 years, SD = 1.1 years) and weighed 18 to 28 kg (mean = 22.9 kg, SD = 2.93 kg). Good health status was confirmed by routine blood testing and clinical examination. To assess the fertility of the dogs, an andrological examination (including physical and ultrasonographic examination and evaluation of semen quality) was performed before the start of the study. Every dog showed sexual interest when exposed to a bitch in estrus.

Dogs were routinely de-wormed and vaccinated. The dogs were housed in private shelters, fed standard commercial dog food twice per day, given water *ad libitum,* and not subjected to changes in habits during the study. Dogs were housed in groups of three in a comfortable primary enclosure with outdoor runs. Indoor space had temperature maintained above 15°C and below 26°C and relative humidity ranging from 30% to 70%.

Investigations were conducted in accordance with the Principles for the Care and Use of Research Animals, promulgated by the European Union. The Italian Ministry of Health (Progetto di Ricerca corrente 2009 IZS SI 11/09: “Randagismo applicazione e valutazione di metodi innovativi per il controllo delle nascite”) approved this study.

### Experimental protocol

At day 0 (T_0_), the animals were randomly assigned to three groups using a random number table: two experimental groups (A and B) of 21 dogs each and a control group (C) of 10 dogs. The first author was aware of group assignment, but technicians collecting data on the subjects were blind to condition. Semen evaluation and collection of blood samples were performed. Subsequently, dogs were lightly sedated with an intramuscular (IM) injection of 5–10 mg of acepromazine maleate (Prequillan, Fatro, Italy) per 10 kg of body weight. The testicular widths were measured with a caliper. According to the scrotal width, the correct dosage of solution was injected into each testicle (see “Preparation and intratesticular injection of CaCl_2_ solution”). Dogs in group A were injected with CaCl_2_ in a solution containing 1% lidocaine chlorhydrate. Dogs in group B were injected with CaCl_2_ in alcohol. Dogs in group C were injected with a saline solution.

At 2, 6, and 12 months (T_1_, T_2_, T_3_, respectively), semen evaluation was performed, and blood samples were taken for testosterone evaluation. At 12 months (T_3_), testicular width was measured. Throughout the trial, the dogs were under clinical observation.

### Preparation and intratesticular injection of CaCl_2_ solution

To prepare the solution containing 20% CaCl_2_ and 1% lidocaine, 20 g of CaCl_2_ dihydrate powder (Sigma Aldrich Corporation) was added to a final volume of 100 mL with a 1% solution of lidocaine chlorhydrate (Salp spa, Italy), mixed, and sterilized in Falcon tubes.

The alcohol solution of 20% CaCl2 dihydrate was prepared as follows: 20 g of CaCl2 dihydrate powder (Sigma Aldrich Corporation) was brought to a final volume of 100 mL of 95% ethanol (Baker Analyzed ACS, JT Baker), mixed, and sterilized in Falcon tubes.

**Figure 1:**
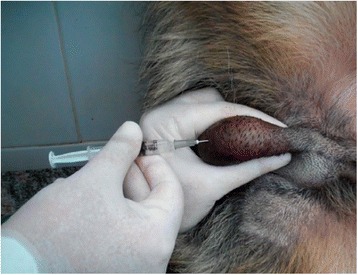
**Intratesticular injection.** Photograph shows procedure of single, bilateral intratesticular injection of 1 mL of 20% CaCl_2_ in ethanol for sterilization of mature male dogs. Each injection was performed using a sterile 22-gauge needle that was directed from the ventral aspect of each testis approximately 0.5 cm from the epididymal tail towards the cranial aspect of that testis. The solution was carefully deposited along the entire route by linear infiltration, while withdrawing needle from proximal to distal end.

The dogs received a single, bilateral intratesticular injection of solution (Figure 1) proportional to testicular width: animals with scrotal diameters of 19–22 millimeters (mm) wide received 0.8 mL injections, whereas animals with scrotal diameters of at least 23 mm wide received 1 mL injections [2].

### Semen volume, total sperm count and motility

Semen was collected by digital manipulation of the penis using plastic cones (artificial vaginas) (IMV Technologies, Italia) into sterile graduated tubes at 37°C [2,13,14]. Ejaculate volume was measured to include all three semen fractions obtained. Within 30–60 min semen was examined by computer-assisted sperm analysis (CASA) (IVOS Version 12.2; Hamilton Thorne Biosciences Inc., Beverly, MA, USA), which was validated for a large range of sperm counts [15,16]. Total sperm count and motility were obtained. Results were confirmed by optical microscopy evaluation.

### Assay for serum testosterone

To determine testosterone levels at time intervals T_0_ to T_3_, dogs received subcutaneous (SC) injections of 1,000 international units (I.U.) of human chorionic gonadotropin (hCG) (Creative Biomart, CD, Inc.) [2,17]. At 120 min after the hCG injections, blood was collected as previously described [2]. Testosterone was measured by a chemiluminescence technique (Immulite Immunoassay System, Siemens).

### Routine clinical observations

All the animals were kept under routine clinical observations from T_0_ to T_3_. After the chemical sterilization procedure, continuous observations were conducted for the first 72 hours, followed by daily observations for up to 15 days, followed by observations as indicated by the study protocol. The parameters evaluated during clinical observation included physiological data (respiratory rate, salivation, body weight, appetite, rectal temperature, etc.), response to palpation, posture, vocalization, mental status (submissive, etc.). Behaviors indicative of pain or discomfort, sexual behavior (mounting) and aggressive behavior (growling, snapping) were carefully evaluated [18].

### Measurement of testicular width

Scrotal width was used as an index of testicular size [19]. At T_0_ and T_3_, widths (mm) of the right and left testes were measured using laboratory calipers. Data were expressed as a mean between the width of left and right testicles.

### Statistical analyses

All data were summarized for each individual canine subject by measurement (weight, testosterone level, semen volume, total sperm count, sperm motility, testicular width), group (A, B, C), and time point (T_0_, T_1_, T_2_, T_3_) using the Microsoft Excel 2011 program (Microsoft Corporation, Redmond, Washington, USA). The average of the testicular width measurements were used for analysis. These data were described in terms of the average and standard deviation (SD) and presented as mean ± SD in the results for brevity.

Statistical analyses were conducted using *Statistica* (StatSoft, Inc. Tulsa, OK, USA). Repeated measures of analysis of variance (ANOVA), with Time as the within factor and Group as the between factor, were used to evaluate the measurements in the three groups (A-C) across four time points (T_0_, T_1_, T_2_, T_3_) for testosterone, total sperm count and motility, or two time points (T_0_ and T_3_) for semen volume and testicular width. If the result of the overall test showed significance, then planned comparisons were conducted. Dunnett’s test for comparison to a control group was used, as well as univariate or multivariate planned comparisons to determine if the measures changed after treatment and if the treated groups differed from the control group. A two-tailed significance level of *P* < 0.05 was identified.

## Results

### Routine clinical observation

Before the injection of sterilant or control saline, the mean weight of the dogs was 22.8 ± 2.9 kg. No changes in body weight during the trial were observed. For all dogs, values for hematology and clinical chemistry consistently remained within reference ranges.

**Figure 2:**
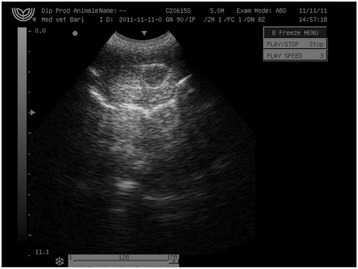
**Scrotal ultrasonography after intratesticular injection of CaCl**_**2**_ **.** A hypoechoic intratesticular area corresponding to a collection of the injected fluid was observed.

All animals in the study tolerated the intratesticular injections of CaCl_2_. Pain parameters did not differ during the study for most dogs. A few dogs, however, showed signs of minor pain at needle puncture of the scrotum during the injection: 2% of dogs injected with either CaCl_2_ in alcohol, lidocaine solution, or normal saline had abdominal muscle contraction, and 1% vocalized. The minor transient discomfort probably was caused by needle puncture of the scrotum or fluid pressure over the testicular capsule. Scrotal ultrasonography (USG) revealed a hypoechoic intratesticular area, corresponding to a collection of the injected fluid (Figure 2).

Even if the injection was performed carefully, seepage occurred in a few dogs. However, the solution was wiped away immediately with dry gauze, and no adverse effects were noticed after the seepage.

During the first two weeks after the CaCl_2_ injection, the dogs in groups A and B and the control dogs (group C) did not experience any agitation, fever, or marked inflammatory swelling of the testis or changes in evaluated parameters. No adverse side effects were noticed at the 2-week period. However, beginning after 24 hours following injection and continuing for the first 3–4 days, a slight increase in firmness of testes on palpation was noticed in dogs in groups A and B and the control dogs (group C); the increased firmness was slightly more noticeable in dogs in group A. From 1 week to approximately 1.5 months in dogs in groups A and B, atrophy of the testes gradually progressed, leaving a small fibrotic remnant. An interference with sexual behavior (i.e., loss of libido, mounting and dominance behavior) and aggression was observed in groups A and B following treatment. In contrast, no testicular changes or alteration of behavior were observed in the control group, C.

### Total sperm count, sperm motility and semen volume

At T_0,_ the mean total sperm count (x10^6^) was 346.2 ± 33.9 in group A, 348.4 ± 32.4 in group B, and 335.9 ± 34.9 in the control group. Analysis of variance procedures indicated a significant interaction of Group and Time for total sperm count (F = 276; *P* < 0.001). Further analyses revealed that this result was due to reduced total sperm count for experimentally treated dogs, but not for the control dogs. No significant variation in total sperm count was noticed at T_1_, T_2,_ and T_3_ in the control group that had received saline injection (F = 1.8; *P* = 0.18) (340 ± 23.3; 313.5 ± 40.5; 311.4 ± 21.4, respectively).

Although total sperm count in the experimental groups A and B did not differ from that of the control group C at baseline T_0_ (F = 0.94; *P* = 0.338), both experimental groups had significantly lower total sperm count than did the controls at T_1_-T_3_ (F = 11476; *P* < 0.001).

In groups A and B, all dogs were azoospermic at T_1_ and T_2_. At T_3_, 17 (81%) dogs in group A were azoospermic, and 4 dogs (19%) were severely oligospermic (40.5 ± 5.1), exhibiting only 5% motility (Table 1). The mean total sperm count in dogs of group A at T_3_ was 7.7 ± 16.4. At T_3_, all dogs of group B were azoospermic. According to statistical analysis, a significant reduction was observed in total sperm counts after intratesticular injection of CaCl_2_ to dogs in group A (F = 2220; p < 0.001) and B (F = 2283; *P* < 0.009) (Table 1).

**Table 1:** **Effects of intratesticular injection of calcium chloride on reproductive parameters at 1 year post-injection**

**Intratesticular Injection**	**Total sperm count (n. × 10** ^**6**^ **)**	**Sperm motility (%)**	**Serum testosterone concentration (ng/dL)**	**Testicular width (mm)**
Saline control (group C)	311.4 ± 21.4 (range 279–342)	80% (range 70–85)	735.2 ± 186.4 (range 398–985)	24.8 ± 2.0 (range 22.5-28.5)
CaCl_2_ in lidocaine (group A)	7.1 ± 16.4 (+) (range 0–47)	5% (range 0–5)	461.1 ± 118.1 (range 225–743)	12.7 ± 1.0 (range 11–15)
CaCl_2_ in alcohol (group B)	0	--	165.7 ± 37.9 (range 104–246)	12.2 ± 0.9 (range 11–15)

Data for effects of single, bilateral intratesticular injection of CaCl_2_ at 1 year post-injection (T_3_) is expressed as the group mean ± standard deviation.

(+) 81% of the dogs remained azoospermic, whereas 19% showed severe oligospermia. (−−) No presence of sperm and, thus, motility, were undetectable.

At T_0_, motility was 90% in group A, 95% in group B, and 80% in the control group (C). Sperm motility in the control group was 80% at all times tested (T_1_-T_3_). Of the 42 treated dogs, only four that were injected with CaCl_2_ in lidocaine had 5% sperm motility (Table 1). Statistical analysis was not possible due to a lack of variability in the data.

Ejaculate volume was not significantly different across groups at T_0_ (Group A: 2.98 ± 0.55; Group B: 3.15 ± 0.68; Group C: 3.45 ± 0.51). Analysis revealed a significant Time by Group interaction (F = 46.2, *P* < 0.001) in which the groups treated with CaCl_2_ had lower semen volume at T_3_ than the control group C (F = 63.5, *P* < 0.001) (Group A: 2.36 ± 0.53; Group B: 1.64 ± 0.55; Group C: 3.50 ± 0.51). Semen volumes in both group A (F = 39.5, *P* < 0.001) and group B (F = 239.9, *P* < 0.001) were significantly reduced from T_0_ to T_3_.

### Assay of serum testosterone

At T_0_, the mean values of testosterone levels (ng/dL) were 456.2 ± 132.4 in group A, 454.6 ± 159.9 in group B, and 721.2 ± 176.2 in the control group (C). For all dogs tested, testosterone values remained within physiological range (100–1000 ng/dL) [17] throughout the course of the study, although a single intratesticular injection of CaCl_2_ was sufficient to decrease plasma testosterone concentrations significantly in the treated dogs. Analyses revealed an overall effect of Group by Time (F = 10.9; *P* < 0.001), with treated groups having lower testosterone after injection than did the control group (F = 165.7; *P* < 0.001). In contrast, changes in the serum testosterone levels in the control group were not statistically significant (*P* > 0.05).

**Figure 3:**
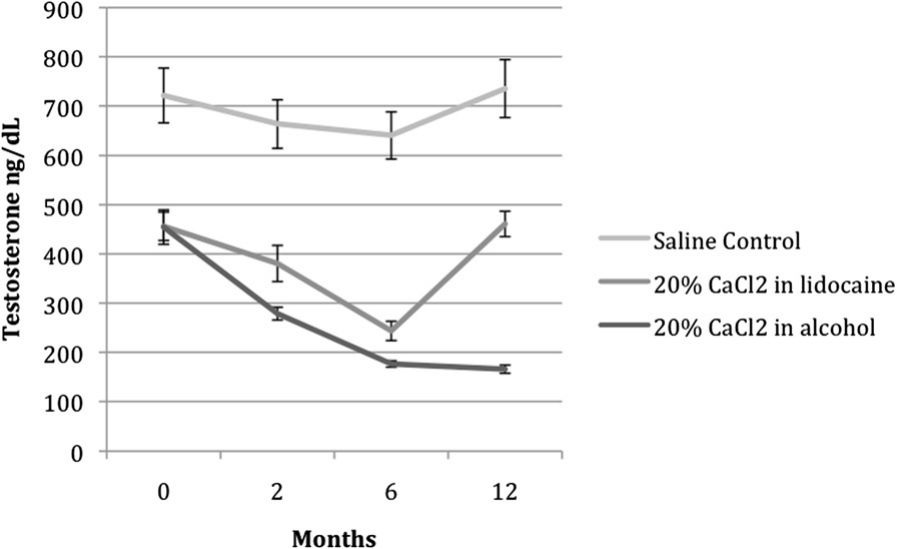
**Effects of intratesticular injection of CaCl**_**2**_ **on serum testosterone levels over time.** Following the injection of CaCl_2_ in lidocaine solution (group A), testosterone decreased significantly (F = 0.47; *P* < 0.003) for up to 6 months, although testosterone levels at 12 months returned to baseline. After injection of calcium chloride in alcohol (group B), testosterone levels decreased significantly (F = 65.1, *P* < 0.001) throughout the 12-month follow-up period.

Figure 3 depicts the levels of serum testosterone graphically over time. Following the injection of CaCl_2_ in lidocaine solution (group A), testosterone decreased significantly for 6 months (F = 0.47; *P* < 0.003), although levels at T_3_ returned to baseline. At 12 months following injection, testosterone levels for the group treated with CaCl_2_ in alcohol (Group B) dropped 63.6%, as compared to baseline. Testosterone levels in group B decreased significantly and remained at the low end of the physiological range throughout the 12-month follow-up period (F = 65.1; *P* < 0.001).

### Measurement of testicular width

Testicular width also varied by Group and Time (F = 412; *P* < 0.001). Average testicular width at baseline (T_0_) was similar across groups. The control group showed no significant difference in testicular width over time (*P* > 0.05).

**Figure 4:**
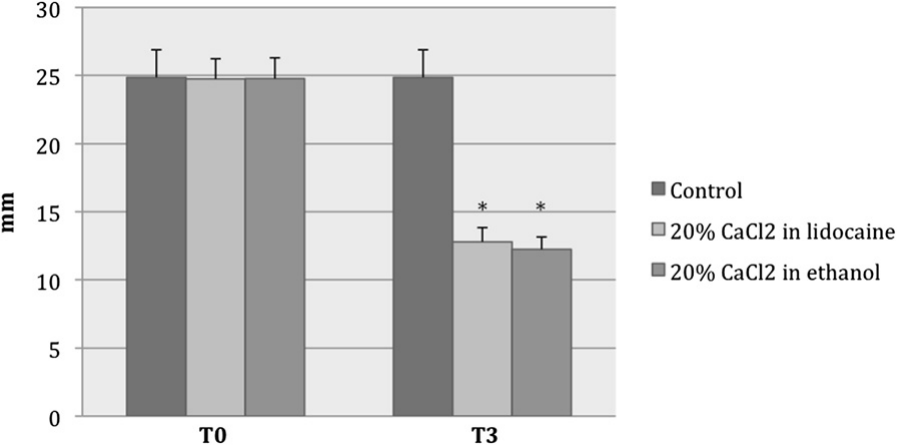
**Changes in testicular width after intratesticular injection of CaCl**_**2**_ **.** At 12 months (T_3_) after treatment with CaCl_2_ (group A and group B), significant reductions in testicular width were observed (*P < 0.001), as compared with no or minimal changes seen in the control (C) group.

At T_0_ versus (*vs*) T_3_, the mean values of testicular width (mm) were 24.7 ± 1.5 *vs* 12.8 ± 1.0 in group A, 24.8 ± 1.5 *vs* 12.2 ± 0.9 in group B, and 24.9 ± 2.1 *vs* 24.9 ± 2.1 in the control group (C) (Figure 4).

After treatment, the width of scrota had declined significantly in both of the CaCl_2_-treated groups (A: F = 2036; *P* < 0.001 and B: F = 2235; *P* < 0.001) and was narrower than that of the control group (F = 802; *P* < 0.001). The average reduction in testicular width at T_3_ was approximately 50% in both group A and group B.

## Discussion

The aim of the current research was to study any potential improvement in the efficacy of using CaCl_2_ as a nonsurgical sterilization method due to the chemical nature of the solvents. In this study, two diluents (lidocaine or alcohol) were tested for use with CaCl_2_ as a sterilant for dogs. Our results indicate that alcohol was a superior solution for CaCl_2_ administration, resulting in complete azoospermia over a 12-month period, decreased sexual behavior, and no side effects.

Alcohol alone is a chemical that causes testicular sclerosis. A study of intratesticular injection of absolute alcohol in rats demonstrated that levels of testosterone were as low as in surgically castrated rats [9]. Studies of ethanol solutions of CaCl_2_ have demonstrated the definite advantages of more consistent efficacy, less pain, and less peripheral inflammation [8].

In our study involving canine male contraception, no or minimal signs of discomfort were observed following injection, with variation dependent on the agent injected. Minor transient pain occurred during the injection, as any needle inserted through skin will cause somatic pain for an instant. The explanation for the relative lack of discomfort following the injection is that afferent nerve endings associated with pain sensation are located on the scrotal skin and in the capsule of the testis, rather than within the testicular and epididymal parenchyma [20]. Given the anatomy of the testes, severe testicular pain when experienced is visceral and triggered by rapid pressure deforming the testicular capsule. During chemical castration, it is important to deliver the injection very slowly to avoid triggering the testicular pressure receptors. In our experience, dogs that had been injected with the alcohol tincture of CaCl_2_ exhibited less discomfort on the day following the injection than those injected with the lidocaine diluent.

Sperm analysis revealed that the injection of CaCl_2_ in alcohol had long-term effectiveness at 1 year post-treatment, whereas the injection of CaCl_2_ in lidocaine solution was effective in all dogs for 6 months. At the 1-year time point, some of the dogs that had been treated with CaCl_2_ in lidocaine solution regained residual production of sperm. However, we cannot affirm that these dogs regained fertility, because the severe oligospermia and poor motility of sperm were unlikely to result in impregnation. Nevertheless it is not possible to exclude that these dogs might regain sufficient sperm production in the future. Regeneration of seminiferous tubules has been reported eight weeks after treatment with 5% concentrations of CaCl_2_, but not at higher dosages [5]. However, our long-term study found that sperm was produced in at least some dogs injected with CaCl_2_ in lidocaine. Thus, our findings differ from reports of short-term studies of similar concentrations of CaCl_2_ that concluded that ‘permanent’ sterilization had occurred [5,6].

Semen volume decreased significantly in dogs injected with CaCl_2_. This was due to a reduction of the second sperm rich fraction of the ejaculate and indicates poor semen quality.

For contraception of stray male dogs, desirable methods require a sufficient reduction in the level of testosterone and, therefore, suppression of sexual behavior. Although previous research on CaCl_2_ in both diluents demonstrated a statistically significant decrease in serum testosterone [5-7], it was not stated explicitly whether the testosterone levels had decreased to below that of physiological range. Prior investigations on the use of CaCl_2_ in lidocaine solution reported the necrotizing properties of CaCl_2_, resulting in low serum concentrations of testosterone [6,7].

In the current study and our previous work [2], a significant decrease in testosterone in all CaCl_2_-treated groups was measured, despite the fact that serum testosterone remained within normal physiological levels over a 12 month period. In the current study, we also observed the disappearance of aggressive and sex-related behavior in the treated dogs throughout the study. To our knowledge, the change in level of testosterone needed to result in a significant decrease in or absence of canine sexual behavior has never been quantified. From the current study, a reduction in testosterone levels to the low end of the physiological range was sufficient to affect behavior. This is important because a reduction in aggression and sexual behavior is usually sought in canine sterilization programs.

The current study is the first to evaluate the long-term effects of different diluents used in CaCl_2_ sterilization. Our findings demonstrate the high potential of 20% CaCl_2_ in alcohol as a sterilant for use in stray male dogs. The sterilant fulfills the principal requirements for application to a population of stray canines. A single, bilateral intratesticular injection for stray dogs is effective in achieving long-term infertility, inhibits sexual behavior, does not cause chronic stress to the animal, causes few inflammatory reactions, lacks other undesirable side effects, is easily performed, and is economical.

## Conclusions

A single, bilateral intratesticular injection of 20% CaCl_2_ in alcohol produced azoospermia in all dogs at one year, representing an optimal method for sterilization in male dogs, whereas the effects of CaCl_2_ in lidocaine solution lasted for only six months. The sterilization approach using CaCl_2_ in alcohol resulted in a durable reduction of testosterone, as compared to baseline levels, and reduced aggressive and sexual behavior. Intratesticular injection of CaCl_2_ in alcohol appears to be an effective and reliable sterilization method in male dogs, making it a good potential alternative to surgical castration. Nevertheless more studies on a larger and more variable population of dogs in a wider weight range as well as in roaming dogs are needed to better understand the applicability of this sterilization method on stray dogs.

## Abbreviations

ANOVA: Analysis of variance
CaCl_2_: Calcium chloride
X g: Centrifugal force X gravity
°C: Centigrade
dL: Deciliter
F: F-statistic (Fisher)
G: Gauge
g: Gram
hCG: Human chorionic gonadotrophin
I.U.: International unit
IM: Intramuscular
kg: Kilogram
x: Magnification
μL: Microliter
mg: Milligram
mm: Millimeter
p: p-value
SD: Standard deviation
SEM: Standard error of the mean
SC: Subcutaneous
vs: Versus

## References

[1] Briggs J: **Non-surgical methods of dog population control – A brief overview of current and future opportunities.** In *Book of Abstracts of the 1*^*st*^*International Conference on Dog Population Management. FERA - Food and Environment Research Agency* York, UK: 2012:21–22.

[2] Leoci R, Aiudi G, Silvestre F, Lissner E, Lacalandra G: **A dose-finding, long-term study on the use of calcium chloride in saline solution as a method of non-surgical sterilization in dogs: Evaluation of the most effective concentration with the lowest risk.** *Acta Vet Scand* 2014, **56:**63. 10.1186/s13028-014-0063-1 PMC4196017 25317740

[3] Koger LM (1977). Calcium chloride, practical necrotizing agent. Bovine Pract.

[4] Koger LM (1978). Calcium chloride castration. Mod Vet Pract.

[5] Samanta PK (1998). Chemosterilization of stray dogs. Indian J Anim Hlth.

[6] Jana K, Samanta PK (2007). Sterilization of male stray dogs with a single intratesticular injection of calcium chloride: a dose dependent study. Contraception.

[7] Jana K, Samanta PK (2011). Clinical evaluation of non-surgical sterilization of male cats with single intra-testicular injection of calcium chloride. BMC Vet Res.

[8] Koger LM: **Calcium chloride, practical necrotizing agent.** In *Proceedings of the Annual Meeting of the American Society of Animal Science.* University of Wisconsin, Madison, Wisconsin, U.S.A.; 1977, **451:**180.

[9] Yoon KJ, Yoon YR (1998). Chemical orchiectomy using absolute alcohol injection into rat testicles. Korean J Urol.

[10] Dixit VP, Lohiya NK, Arya M, Agrawal M (1975). Chemical sterilization of male dogs after a single intra-testicular injection of "Danazol". Folia Biol.

[11] Freeman MD, Coffey DS (1973). Sterility in male animals induced by injection of chemical agents into the vas deferens. Fertil Steril.

[12] Ellingboe J, Varanelli CC (1970). Ethanol inhibits testosterone biosynthesis by direct action on Leydig cells. J Lipid Res.

[13] Freshman JL (2002). Semen collection and evaluation. Clin Technol Small Anim Pract.

[14] Kutzler MA (2005). Semen collection in the dog. Theriogenology.

[15] Leoci R, Aiudi G, De Sandro Salvati A, Silvestre F, Binetti F, Lacalandra GM (2009). Ultrasound as a mechanical method for male dog contraception. Reprod Dom Anim.

[16] Rijsselaere T, Van Soom A, Maes D, Nizanski W (2012). Computer-assisted sperm analysis in dogs and cats: An update after 20 years. Reprod Dom Anim.

[17] Santana M, Batista M, Alamo D, Cabrera F, Gonzalez F, Gracia A (2012). Influence of sexual stimulation and the administration of human chorionic gonadotropin on plasma testosterone levels in dogs. Reprod Dom Anim.

[18] Hansen BD (2003). Assessment of pain in dogs: Veterinary clinical studies. ILAR J.

[19] Woodall PF, Johnstone IP (2008). Scrotal width as an index of testicular size in dogs and its relationship to body size. J Small Anim Pract.

[20] Kutzler M, Wood A (2006). Non-surgical methods of contraception and sterilization. Theriogenology.

